# Global Analysis of Public Cheese Microbiomes Reveals Widespread Bacterial Taxa and Geographic Community Structure

**DOI:** 10.3390/ijms27219798

**Published:** 2026-10-31

**Authors:** Jan Sadurski, Adam Staniszewski, Elif Bircan Muyanlı, Remziye Yılmaz, Adam Waśko

**Affiliations:** 1 Department of Biotechnology, Microbiology and Human Nutrition, Faculty of Food Science and Biotechnology, University of Life Sciences in Lublin, Skromna 8, 20-704 Lublin, Poland; adam.wasko@up.edu.pl; 2 Department of Invertebrate Ecophysiology and Experimental Biology, University of Life Sciences in Lublin, Doświadczalna 50A, 20-280 Lublin, Poland; adam.staniszewski@up.edu.pl; 3 FoodOmics Laboratory, Department of Food Engineering, Hacettepe University, 06800 Ankara, Turkey; elif.bircan.ebm@gmail.com (E.B.M.); remziye@hacettepe.edu.tr (R.Y.)

**Keywords:** cheese microbiome, metagenomics, microbiome, microbial diversity, bioinformatics, whole-genome sequencing, 16S rRNA sequencing, microbial ecology, public datasets, meta-analysis

## Abstract

Cheese microbiomes remain difficult to compare globally because public sequencing datasets are fragmented and methodologically heterogeneous. Here, we curated 14,203 sequencing records from 233 BioProjects and retained 8910 records from 112 BioProjects for standardized reanalysis, establishing a global comparative framework for cheese microbiome research. Three taxonomic profiling pipelines (QIIME2, Kraken2 and mOTUs) recovered broadly consistent genus-level patterns dominated by lactic acid bacteria, although quantitative concordance varied between methods and study collections. Whole-genome shotgun analysis identified 12 widespread bacterial species independently detected in at least 50% of BioProjects by both Kraken2 and mOTUs, with *Lactococcus lactis*, *Streptococcus thermophilus* and *Lactococcus cremoris* detected in at least 84% of BioProjects in both analyses. Geographic structure remained evident after accounting for dominant community composition: sample-origin country explained 36.6% of community variation in the primary analysis and remained associated with community composition in balanced subsampling and analyses removing or continuously adjusting for five dominant lactic acid bacterial genera. However, public datasets were strongly geographically imbalanced, with European BioProjects dominating the available collection. Together, these results establish a reproducible framework and comparative baseline for investigating cheese microbiome diversity, geographic structure and cross-study patterns across globally distributed public sequencing datasets.

## 1. Introduction

Cheese is one of humanity’s oldest fermented foods and represents an ecologically complex, highly selective microbial ecosystem [1]. For millennia, dairy fermentations have transformed highly perishable milk into stable, nutrient-dense products with extended shelf-life and substantial economic and cultural value [2]. These biochemical transformations are driven by complex successions of bacteria, yeasts, and filamentous fungi that dictate acidification, proteolysis, lipolysis, and the synthesis of key volatile compounds [3]. Traditional lactic acid bacteria (LAB), including members of the genera *Lactococcus*, *Streptococcus*, *Lactobacillus*, *Lacticaseibacillus* and *Leuconostoc*, act as primary fermenting agents [4,5], while secondary microbiota from raw milk, production environments, and ripening facilities drive the unique sensory diversification of mature cheeses [6].

During manufacture and ripening, these colonizing communities are repeatedly exposed to severe, recurring physicochemical stressors including rapid lactic acid production leading to low pH, high osmotic pressure from salting, moisture depletion, and intense microbial competition favoring a restricted set of highly adapted taxa. Consequently, cheese microbiomes represent a delicate balance between ecological convergence imposed by deterministic environmental filters and diversification driven by local biological, technological, and geographical factors [6]. Determining how these processes interact on a global scale is fundamental to understanding whether cheeses produced across different regions share a common microbial structure or represent independent, isolated ecosystems.

Over the past decade, high-throughput sequencing has revolutionized dairy microbiology by enabling cultivation-independent characterization of food-associated communities at species, strain, and functional levels [7]. Pioneering metagenomic investigations have successfully linked microbial succession to flavor formation [8,9], mapped phage-host dynamics in cheese rinds [10], and dramatically expanded genomic catalogs [11]. However, despite rapid progress in describing individual cheese ecosystems, a fundamental scientific gap persists: cheese microbiome data remain highly fragmented [12]. Because sequencing datasets have accumulated through independent, localized studies focused on specific cheese varieties or regions, the resulting resources are dispersed across public repositories under highly heterogeneous annotation standards [12,13].

Furthermore, substantial technical noise is introduced by differences in raw materials, DNA extraction protocols, PCR primers, sequencing platforms (although Illumina dominates at 90.3%) [14], and taxonomic profiling pipelines [14,15]. This methodological fragmentation makes it impossible to distinguish true, reproducible biological signals from technical artifacts, creating a critical barrier to the industrial, regulatory, and translational application of food metagenomics.

To resolve this limitation and transition food metagenomics from localized descriptions to a predictive science, we must move beyond comparing disparate, published taxonomic profiles and instead perform a standardized, harmonized reanalysis of the raw sequencing data [7]. Here, we systematically retrieved 14,203 raw cheese-associated sequencing records from the NCBI Sequence Read Archive (SRA) representing 233 BioProjects [9,16] and, following rigorous quality-control and BioProject-level curation, harmonized 8910 high-quality records from 112 independent BioProjects spanning both amplicon and whole-genome shotgun (WGS) sequencing [11,16]. We reanalyzed the underlying raw data using three independent bioinformatic pipelines representing fundamentally distinct methodologies: QIIME2 for amplicon datasets (denoising short 16S rRNA sequences using DADA2) [17,18], Kraken2 (k-mer-based classification of WGS reads) [18,19], and mOTUs (marker-gene-based profiling of WGS data) [19,20].

The true novelty of this study lies not merely in assembling the largest harmonized food-associated metagenomic dataset to date, nor in the technical benchmarking of three independent taxonomic profiling pipelines. Rather, the defining innovation of this work is the integration of these high-throughput resources to establish the first comprehensive global reference framework for cheese microbiome research. Within this unified scientific narrative, the standardized reanalysis of public sequencing data, the identification of a globally conserved microbial core, the characterization of recurrent starter-community archetypes, and the systematic exposure of metadata reporting limitations are not isolated analytical steps. Instead, they represent interconnected pillars of a singular, overarching vision: to transition food metagenomics from localized, descriptive studies toward a robust, comparative global science capable of guiding future dairy innovation and authentication [8,21].

The defining innovation of this study is the integration of large-scale public sequencing resources within a standardized analytical framework for comparative cheese microbiome research. Rather than relying on previously published taxonomic profiles, we reanalyzed public sequencing data using harmonized workflows to identify recurrent taxonomic patterns, evaluate geographic community structure, assess cross-pipeline concordance, and quantify limitations in the geographic and metadata coverage of currently available datasets. This framework enables biological patterns shared across heterogeneous public studies to be distinguished more clearly from methodological and study-level variation.

Within this framework, our study addresses four main objectives:

1. First, we investigated whether recurrent cheese-associated bacterial taxa can be identified across heterogeneous public datasets and whether widespread taxa persist at species-level resolution in independently generated WGS profiles.
2. Second, we evaluated whether microbial community composition is associated with BioSample-reported sample-origin country among the studies represented in the public dataset. Because geographic origin, cheese type, production practices, laboratory effects, and other study-level factors cannot be fully separated using available metadata, geographic associations were interpreted conservatively and evaluated using balanced and composition-adjusted sensitivity analyses.
3. Third, we assessed the consistency of taxonomic patterns across analytical approaches. Kraken2 and mOTUs, applied to the same WGS collection, enabled direct cross-pipeline comparison, whereas comparisons with QIIME2 were used to evaluate aggregate genus-level patterns across independently assembled amplicon and WGS study collections.
4. Fourth, we characterized the geographic representation, metadata completeness, and analytical limitations of the public cheese microbiome sequencing landscape and established a reproducible resource comprising curated metadata, processed abundance matrices, source data, and analysis workflows for future comparative studies.

Together, these analyses establish a global comparative framework for investigating widespread bacterial taxa, geographic community structure, and cross-study taxonomic patterns in public cheese microbiome datasets, while explicitly accounting for the methodological and metadata limitations inherent to retrospective analyses of heterogeneous sequencing studies.

## 2. Results

### 2.1. Public Cheese Microbiome Repositories Remained Geographically Unbalanced

A comprehensive search of the NCBI Sequence Read Archive (SRA), performed on 7 February 2026, identified 14,203 cheese-associated sequencing records representing 233 BioProjects. Following metadata-based filtering, BioProject-level curation, and manual quality assessment, 8910 SRA records from 112 BioProjects were retained (Table 1), corresponding to 62.7% of the original sequencing records and 48.1% of the identified BioProjects. Of these, 8100 SRA records from 92 BioProjects were assigned to the amplicon subset and 810 records from 21 BioProjects to the WGS subset; one BioProject contained both sequencing strategies. A complete list of retained BioProjects and associated metadata is provided in Supplementary Table S1.

**Figure 1:**
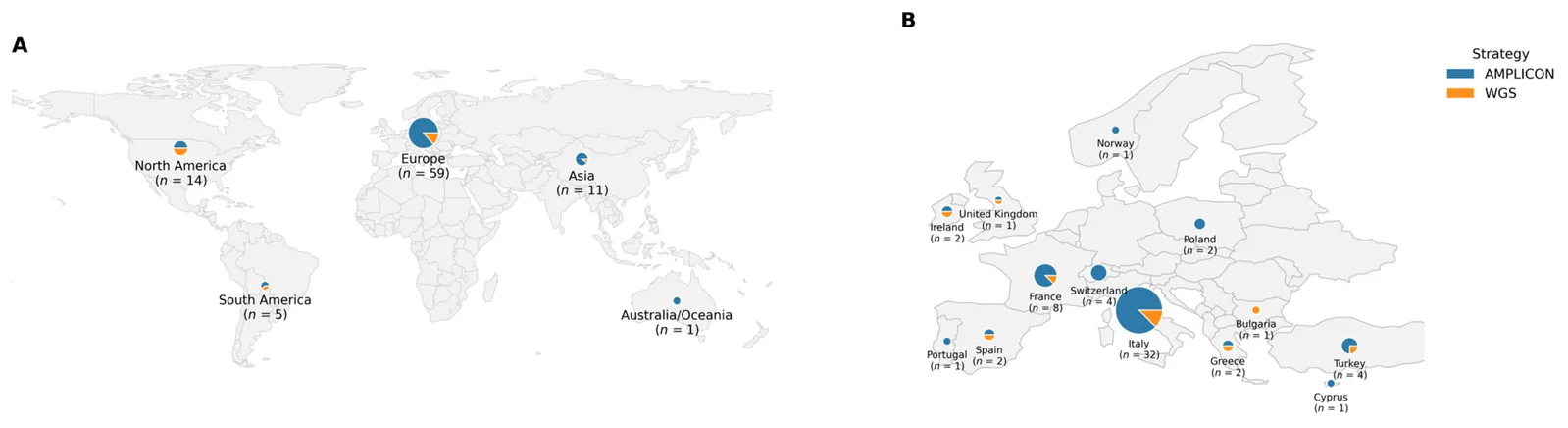
Geographic distribution of public cheese-associated microbiome datasets retained for analysis based on BioSample-reported sample origin. (**A**) Global distribution of BioProjects by continent. (**B**) European distribution by sample-origin country. Pie-chart segments indicate the number of BioProjects represented by amplicon and WGS data, respectively. The geographically resolved dataset comprised 7592 retained SRA records belonging to 89 unique BioProjects across 21 countries (73 BioProjects represented by amplicon data and 17 by WGS data; one BioProject was represented in both sequencing strategies). BioProjects containing samples from more than one country contribute to each corresponding geographic group; therefore, geographic and sequencing-strategy counts are not additive estimates of the total number of unique BioProjects.

Geographic representation was subsequently reassessed using BioSample-reported sample-origin metadata rather than sequencing-center location. Among BioProjects for which microbiome profiles were successfully generated and sample-origin information was available, 89 unique BioProjects comprising 7592 retained SRA records could be geographically resolved, representing 21 countries (Figure 1; Supplementary Table S4). Of these, 73 BioProjects were represented by amplicon data and 17 by WGS data, with one BioProject represented by both sequencing strategies. Two WGS BioProjects contained samples originating from more than one country.

Geographic coverage remained strongly uneven, with European countries contributing a substantial proportion of the available datasets, whereas several world regions were represented by comparatively few BioProjects. Amplicon BioProjects were generally larger than WGS BioProjects (median 24 versus 16 SRA records), and the two largest amplicon studies alone contributed 4588 SRA records. This marked imbalance in the number and size of geographically represented BioProjects motivated the use of restricted and balanced sensitivity analyses in subsequent geographic comparisons.

### 2.2. A Conserved Cheese Microbiota Emerged Despite Heterogeneous Public Datasets

**Figure 2:**
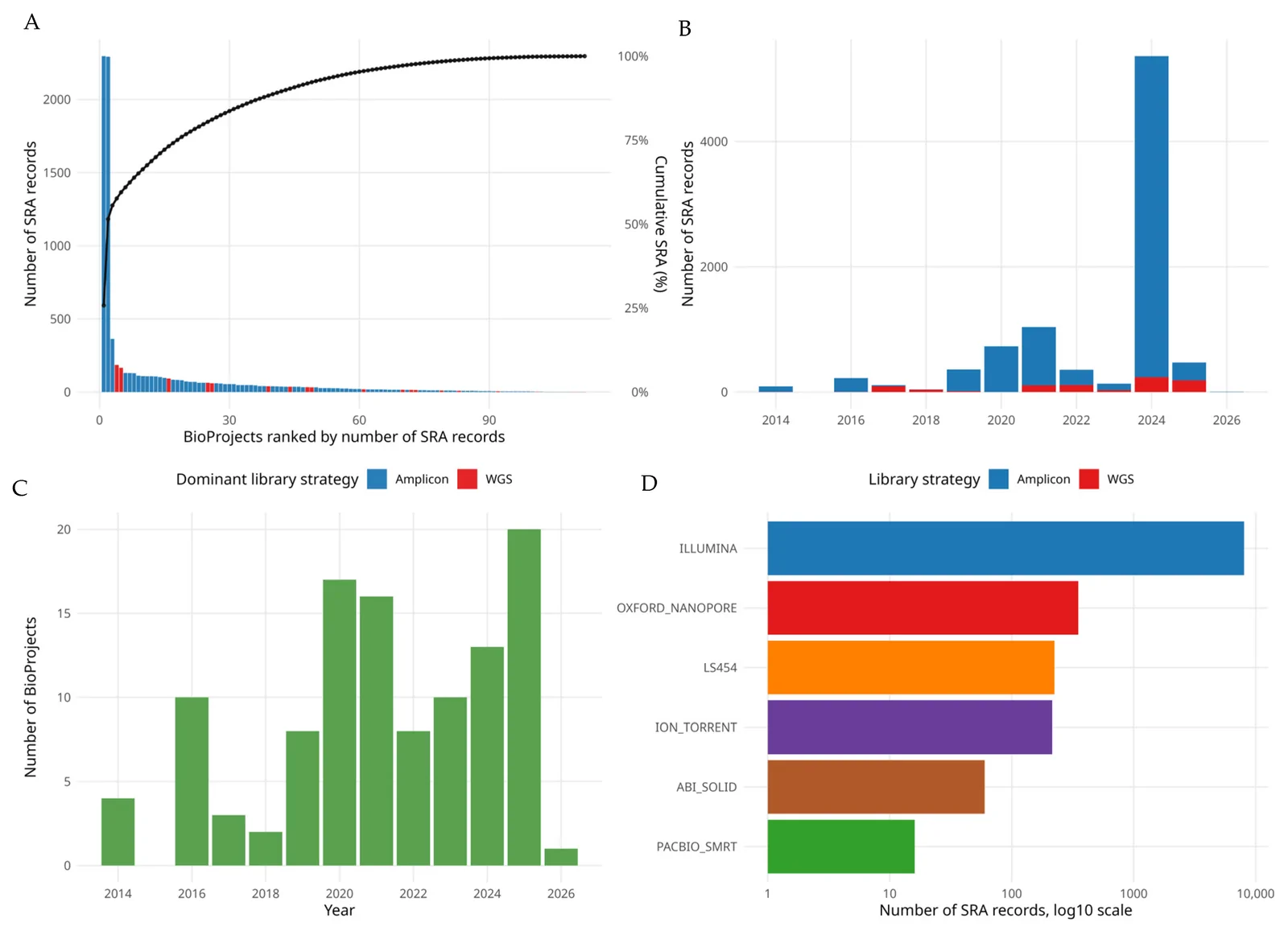
Temporal and technical structure of the public cheese microbiome dataset. (**A**) BioProjects ranked by the number of associated SRA records; the cumulative line shows the cumulative proportion of records represented by successively ranked projects. (**B**) Number of SRA records by year and sequencing strategy (amplicon and WGS). (**C**) Number of BioProjects represented in each year. (**D**) Number of SRA records generated using individual sequencing platforms, shown on a logarithmic scale.

The curated dataset showed substantial heterogeneity in study size, deposition year, and sequencing technology (Figure 2). More than half of all analyzed SRA records originated from only two BioProjects, whereas the 25 largest projects contributed over 80% of the complete dataset (Figure 2A). Public deposition increased rapidly after 2020 and peaked in 2024 with 5363 SRA records (Figure 2B), while the annual number of newly deposited BioProjects remained comparatively stable (Figure 2C). Illumina sequencing accounted for 90.3% of all datasets (Figure 2D).

**Figure 3:**
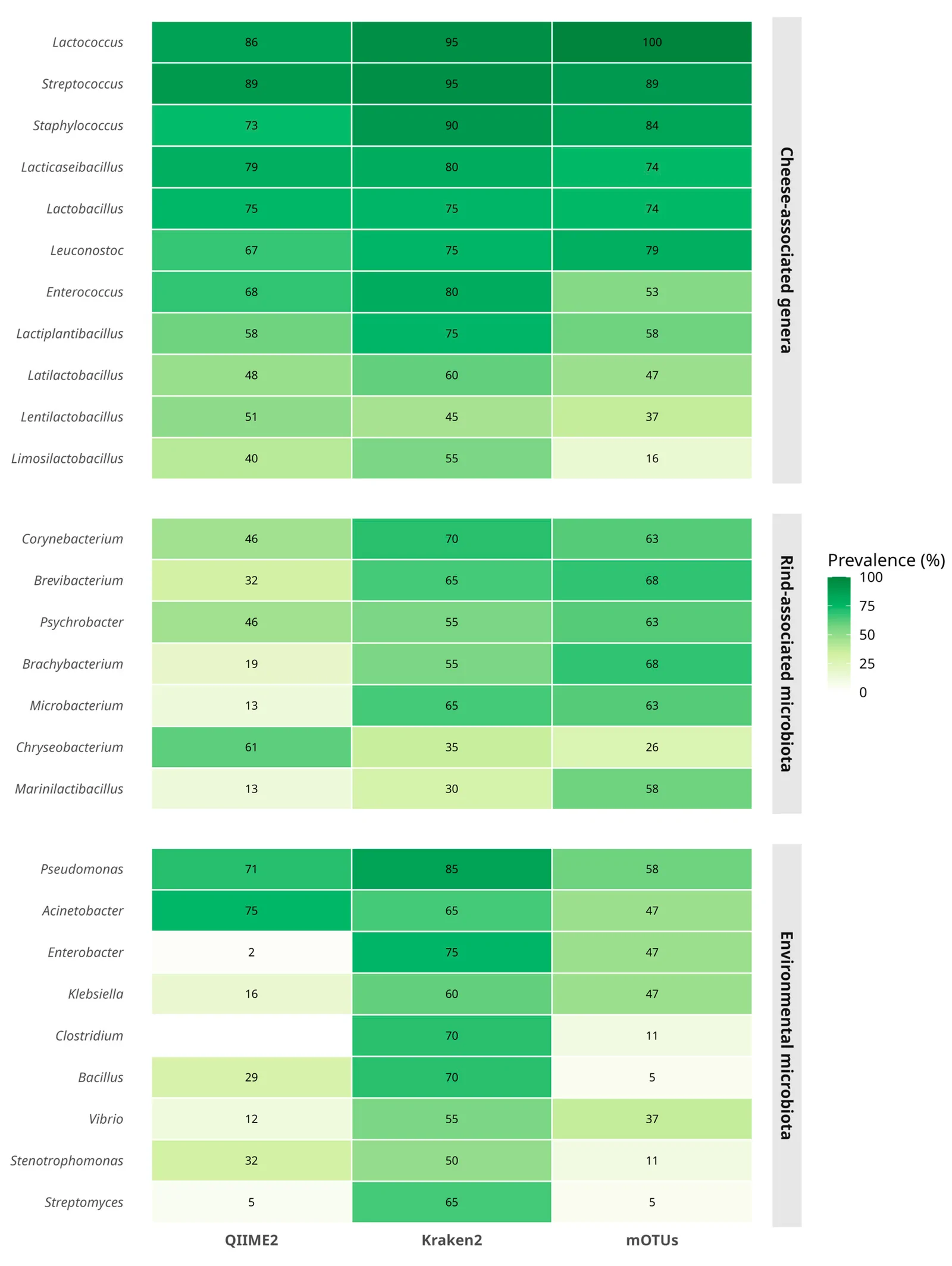
Cross-method prevalence of dominant bacterial genera in public cheese microbiome datasets. Heatmap showing the prevalence of dominant bacterial genera across QIIME2, Kraken2 and mOTUs profiles. Prevalence was calculated as the proportion of BioProject-level matrix rows in which a genus was detected above the 0.01% relative-abundance threshold, using 86 QIIME2, 21 Kraken2 and 20 mOTUs matrix rows, respectively. Genera are arranged into predefined visualization groups representing cheese-associated genera, rind-associated microbiota and environmental microbiota. Blank cells correspond to 0% prevalence, indicating that the genus was not detected above the applied threshold in any BioProject-level matrix row for that pipeline.

Despite this marked heterogeneity in study design, sequencing technology, and dataset composition, all three analytical pipelines (QIIME2, Kraken2, and mOTUs) recovered broadly similar patterns among dominant bacterial genera (Figure 3). Across the three approaches, cheese microbiomes were frequently represented by *Lactococcus*, *Streptococcus*, *Lactobacillus*, *Lacticaseibacillus*, *Leuconostoc* and *Staphylococcus*. Surface-ripened cheese-associated genera, including Brevibacterium, Corynebacterium, Brachybacterium and Psychrobacter, were also detected, although generally at lower prevalence.

Concordance was strongest between Kraken2 and mOTUs, which were evaluated on the same set of 19 WGS BioProjects. Across the harmonized and abundance/prevalence-filtered genus set, Kraken2–mOTUs correlations were ρ = 0.513 for BioProject-level prevalence and ρ = 0.654 for mean relative abundance. In contrast, comparisons involving QIIME2 represented aggregate taxonomic-pattern comparisons across independently assembled amplicon and WGS collections rather than paired estimates of pipeline agreement. Corresponding prevalence and abundance correlations were ρ = 0.361 and 0.482 for QIIME2–Kraken2 and ρ = 0.258 and 0.417 for QIIME2–mOTUs, respectively (Figure 3; Supplementary Tables S2 and S3). Collectively, these analyses indicated that recurrent dominant cheese-associated bacterial genera were recovered across the three profiling approaches, while the strength of quantitative concordance depended on both the analytical pipeline and the underlying study collection.

### 2.3. Geographic Variation Extended Beyond Starter-Community Composition

**Figure 4:**
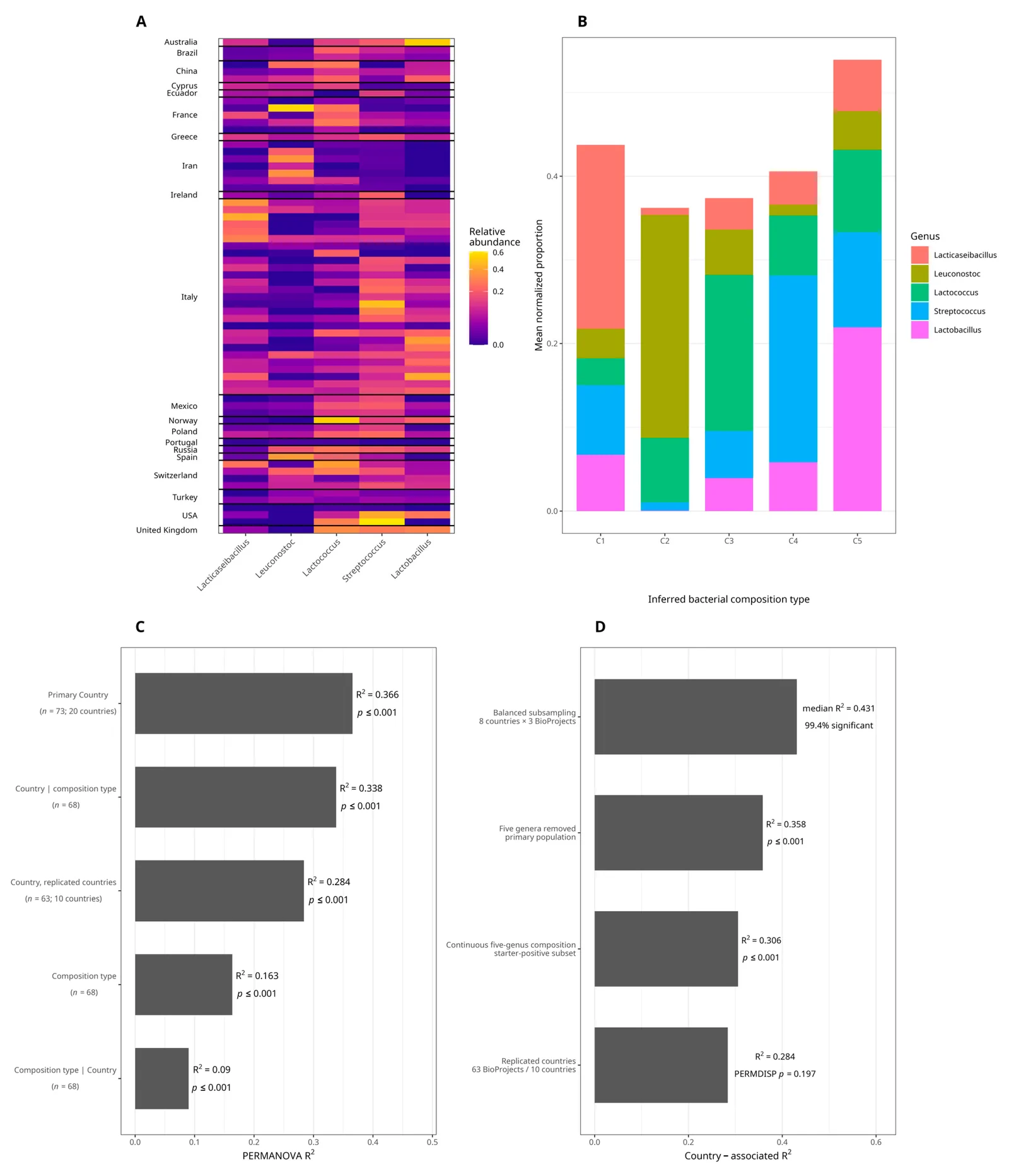
Geographic and compositional structure of cheese microbiomes across BioProjects. (**A**) Relative abundances of five dominant composition-associated genera across BioProjects. BioProjects are ordered by BioSample-reported sample-origin country, then by descriptive composition type (C1–C5), and finally by BioProject accession; horizontal separators delineate countries, and the adjacent color strip indicates the composition type assigned to each BioProject. (**B**) Five descriptive composition types derived from the five-genus profiles. (**C**) PERMANOVA effect sizes (R^2^) for country and composition type, including sequential models. (**D**) Sensitivity analyses of the country association using replicated-country filtering, removal or continuous adjustment of the five genera, and balanced subsampling. Full statistical results are provided in Supplementary Tables S5–S7.

We next examined whether geographic differences in cheese microbiome composition persisted after accounting for variation in dominant lactic acid bacteria. Geographic analyses were restricted to 73 BioProjects with a single BioSample-reported sample-origin country, representing 20 countries. In the primary PERMANOVA, country was significantly associated with community composition (R^2^ = 0.366, pseudo-F = 1.608, *p* = 0.001; Figure 4C; Supplementary Table S5). However, multivariate dispersion also differed among countries (PERMDISP F = 4.484, *p* = 0.001), indicating that the primary geographic association may have reflected a combination of differences in community location and within-country dispersion.

To assess the influence of unequal geographic representation, we repeated the analysis after restricting the dataset to countries represented by at least two BioProjects. In this subset (63 BioProjects from 10 countries), the country association remained significant (R^2^ = 0.284, pseudo-F = 2.334, *p* = 0.001), whereas differences in multivariate dispersion were not significant (PERMDISP F = 1.425, *p* = 0.197; Figure 4D; Supplementary Table S5). We further performed 1000 balanced subsampling iterations using eight countries represented by at least three BioProjects and randomly selecting three BioProjects per country in each iteration. The median country-associated R^2^ was 0.431 (empirical 2.5–97.5% interval, 0.378–0.500); 99.4% of iterations yielded PERMANOVA *p* ≤ 0.05, while none yielded PERMDISP *p* ≤ 0.05 (Supplementary Table S7). These sensitivity analyses indicated that the geographic association was retained when differences in the number of BioProjects per country were reduced.

We next examined the contribution of five dominant composition-associated genera (*Lactococcus*, *Streptococcus*, *Lacticaseibacillus*, *Lactobacillus* and *Leuconostoc*). Among 68 BioProjects containing these genera, k-means partitioning into five groups was used as a descriptive representation of contrasting community compositions rather than as evidence for five discrete technological or starter-defined categories (Figure 4A,B). Evaluation across k = 2–10 did not identify k = 5 as a uniquely optimal solution (Supplementary Table S6). Accordingly, these groups were described as descriptive composition types and were not interpreted as independent metadata on starter-culture use.

Within this 68-BioProject subset, country and composition type were both associated with microbiome variation. In sequential PERMANOVA models, country accounted for R^2^ = 0.338 after composition type was entered first, whereas composition type accounted for R^2^ = 0.090 after country was entered first (both *p* = 0.001; Figure 4C; Supplementary Table S5). A descriptive decomposition of these sequential model results corresponded to 33.8% country-associated variation, 9.0% composition-type-associated variation, 7.3% variation shared between the two terms, and 49.9% residual variation. Because composition types were derived directly from the microbiome profiles and the sequential fractions depend on model term order, these values were interpreted as statistical associations within the dataset rather than as independent causal effects of geography or production technology.

Finally, the country association persisted in two complementary sensitivity analyses that did not depend on the five-group classification. After removing *Lactococcus*, *Streptococcus*, *Lacticaseibacillus*, *Lactobacillus* and *Leuconostoc* from the community matrix, country remained significantly associated with composition across the 73 BioProjects (R^2^ = 0.358, pseudo-F = 1.559, *p* = 0.001). Similarly, after including the relative contributions of these genera as continuous composition covariates, the additional country-associated fraction remained significant in the 68-BioProject subset (R^2^ = 0.306, pseudo-F = 1.558, *p* = 0.001; Figure 4D; Supplementary Table S5). Together, these analyses supported a robust association between BioSample-reported sample-origin country and cheese microbiome composition, while indicating that the observed geographic structure could not be attributed solely to variation in the five dominant composition-associated genera.

### 2.4. Widespread Cheese-Associated Taxa Extended to Species-Level Resolution

**Figure 5:**
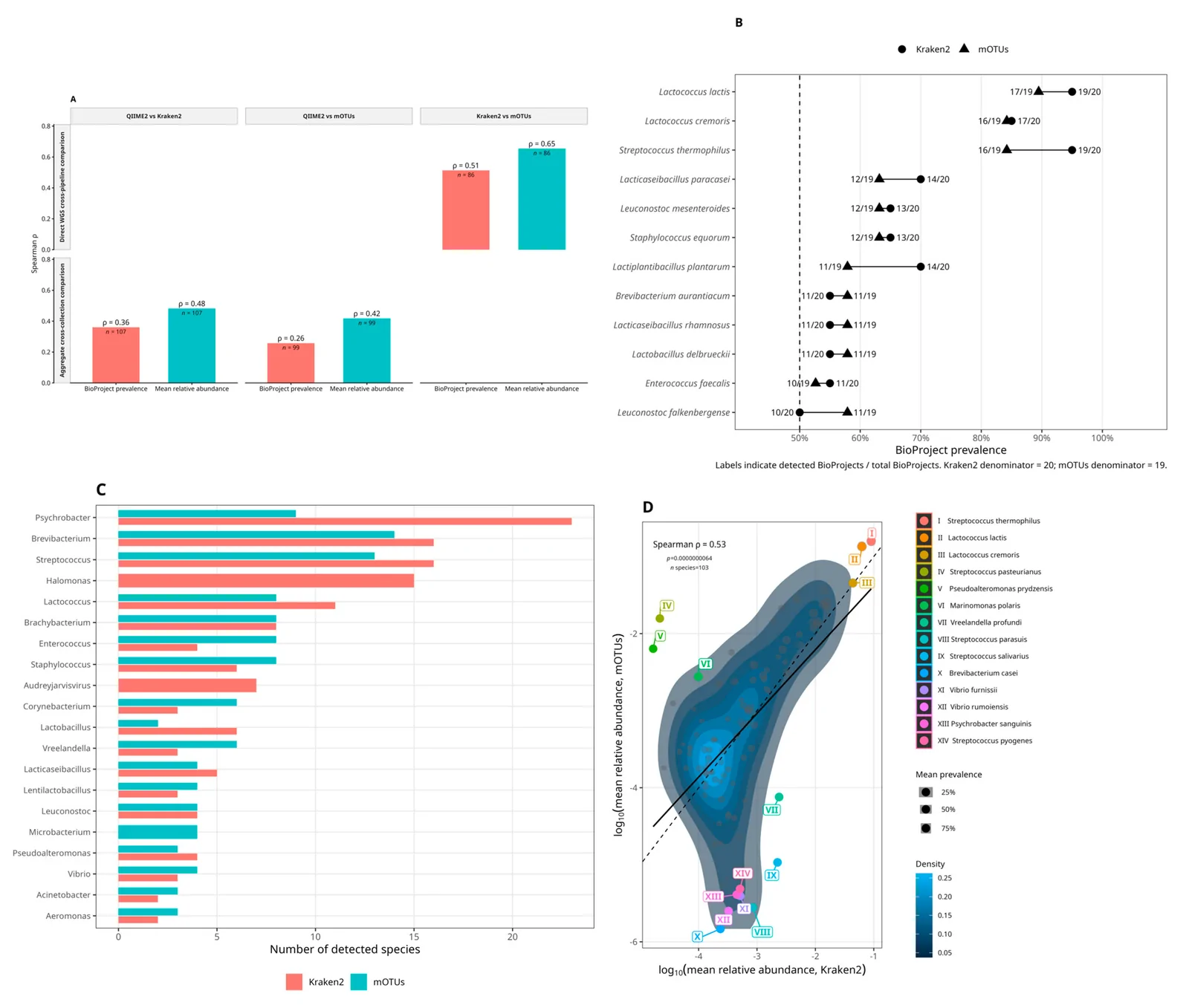
Comparison of species-level and genus-level taxonomic patterns across profiling pipelines. (**A**) Genus-level concordance in BioProject prevalence and mean relative abundance, with Kraken2–mOTUs representing direct WGS pipeline concordance and QIIME2 comparisons representing aggregate patterns across independent datasets. (**B**) Widespread WGS bacterial species detected by both Kraken2 and mOTUs in ≥50% of BioProjects, with highly widespread species (≥75%) indicated. (**C**) Species richness within the dominant bacterial genera identified by Kraken2 and mOTUs. (**D**) Mean abundance concordance between Kraken2 and mOTUs across 103 shared species retained after prevalence and abundance filtering.

We finally examined whether widespread cheese-associated taxa could also be identified at species-level resolution using WGS datasets. Species-level analyses focused on Kraken2 and mOTUs, which were applied to the same WGS collection, enabling direct comparison of taxonomic patterns between the two profiling pipelines (Figure 5A).

Among 448 species detected by both WGS pipelines, 12 met the primary definition of widespread species, requiring independently estimated BioProject prevalence ≥50% and mean relative abundance ≥0.01% in both Kraken2 and mOTUs (Figure 5B; Supplementary Table S8). *L. lactis*, *S. thermophilus* and *L. cremoris* were the most widespread species, each occurring in ≥84% of BioProjects in both analyses. Eleven of the 12 widespread species remained above the 50% prevalence threshold in all leave-one-BioProject-out analyses (Supplementary Table S10), indicating that the widespread set was largely insensitive to individual BioProjects. The number of species classified as widespread varied across alternative prevalence and detection thresholds (Supplementary Table S9), demonstrating the expected dependence of core-set size on the operational thresholds used. Several dominant genera comprised multiple species rather than a single representative taxon, particularly *Brevibacterium*, *Psychrobacter*, *Streptococcus* and *Lactococcus* (Figure 5C).

Across 103 shared species retained after prevalence and abundance filtering, mean relative abundances estimated by Kraken2 and mOTUs were moderately correlated (ρ = 0.534, *p* = 6.36 × 10^−9^; Figure 5D). This comparison represented concordance of across-BioProject mean species abundances rather than sample-level classification accuracy. Together, these results identified a robust set of widespread bacterial species across public cheese WGS datasets while also demonstrating that quantitative agreement between taxonomic profiling pipelines remained incomplete at species-level resolution.

## 3. Discussion

This comparative reanalysis reveals two complementary features of public cheese-associated microbiomes: a recurrent bacterial component shared across heterogeneous studies and country-associated community variation extending beyond five dominant fermentation-associated genera. Together, these findings connect the common ecological requirements of cheese fermentation with the diversity of regional production systems. The recurrence of dominant bacteria is consistent with established descriptions of cheese microbial communities [3,22] and with ecological filtering during fermentation [20,23]. The manufacture of cheese subjects colonizing microorganisms to a sequence of physicochemical stressors: rapid lactic acid production leading to low pH, high osmotic pressure from dry salting or brining, moisture depletion, nutrient restriction, and intense microbial competition [8,17,21,22].

These conditions favor microorganisms adapted to dairy fermentation and help explain the repeated recovery of *Lactococcus*, *Streptococcus*, *Lactobacillus*, *Lacticaseibacillus* and *Leuconostoc* [21,22]. Their recurrence across independently generated datasets supports the view that shared fermentation conditions constrain the range of bacterial communities commonly established in cheese-associated systems. Ecological selection during fermentation provides a plausible mechanism for this convergence [20,23], while the differences retained among studies show that these constraints accommodate substantial compositional diversity.

The species-level results strengthen this interpretation. Twelve bacterial species satisfied the prevalence and abundance criteria independently in both WGS pipelines, and *L. lactis*, *S. thermophilus*, and *L. cremoris* occurred in at least 84% of BioProjects in both analyses. These three species form the most consistently recovered component of the WGS collection. Moreover, eleven of the twelve widespread species retained their classification in every leave-one-BioProject-out iteration, showing that their inclusion was not dependent on any single study. *Leuconostoc falkenbergense* was the exception: its classification was sensitive to project exclusion because its Kraken2 prevalence lay exactly at the primary threshold. The sensitivity analyses therefore distinguish a stable set of widespread bacteria from taxa positioned near the operational boundary (Supplementary Tables S8–S10).

Alongside environmental selection, the long history of human management of dairy fermentations provides a complementary explanation for recurrent bacterial composition [1,5]. For generations, back-slopping (inoculating a new batch with a portion of a successful previous fermentation) has favored resilient, fast-acidifying strains [1,6]. In industrial dairy production, this selection has been formalized into commercial starter cultures [6]. Repeated microbial introduction and selection during production offer a biological context for the contrasting profiles of dominant fermentation-associated genera observed here [21,24]. The five descriptive composition types summarize these contrasts, capturing variation in the relative contributions of *Lactococcus*, *Streptococcus*, *Lacticaseibacillus*, *Lactobacillus* and *Leuconostoc*. Their value lies in organizing compositional variation across studies rather than identifying five discrete starter formulations: k = 5 was not uniquely optimal, and actual inoculation records were not consistently available.

The recurrent fermentation-associated component coexists with a broader bacterial assemblage, consistent with the ecological diversity of cheese systems [21]. While commercial starter cultures are intentionally inoculated to drive the primary fermentation, they can be complemented by microorganisms from raw milk, facility-associated microbial reservoirs, and regional ripening environments [24,25,26,27,28,29]. Interactions among these microorganisms contribute to community succession and differentiation during production and ripening [24,25,26]. This provides a useful ecological interpretation of our combined findings: shared fermentation-associated taxa account for recurrent features of cheese microbiomes, while variation in their proportions and in the accompanying community contributes to differentiation among studies. Comparable regional and strain-level differentiation has also been reported across naturally fermented dairy ecosystems, indicating that substantial microdiversity may persist beneath apparently conserved higher-level community structures [4,30,31].

A central finding is that country-associated community variation persisted across several complementary analytical settings. Country accounted for 36.6% of variation in the primary analysis of 73 single-country amplicon BioProjects. Restricting the analysis to countries represented by at least two BioProjects retained the association, and balanced subsampling produced significant PERMANOVA results in 99.4% of iterations. Unlike the primary analysis, these restricted and balanced comparisons did not detect significant differences in multivariate dispersion. The persistence of the association under reduced group-size imbalance strengthens the evidence that the geographic pattern is not confined to the original, unevenly represented collection (Supplementary Tables S5 and S7).

The association also remained after removing the five dominant composition-associated genera and after accounting for their continuous relative contributions. This is biologically informative because it places country-associated differentiation beyond variation in the dominant fermentation-associated component alone. Regional and production-associated microbial patterns reported in cheese studies provide a context for this result [2,4,32,33,34]. Country can encompass differences in raw materials, cheese varieties, manufacturing practices, and production environments; the present analysis shows that the combined variation associated with these geographic groupings remains detectable across independent studies. It does not isolate geography as a causal mechanism, but establishes a persistent association that more detailed, metadata-rich studies can resolve into its biological and technological components.

The use of complementary profiling approaches provided a second perspective on the reproducibility of taxonomic patterns in public data. Differences in profiling principles and reference resources are central considerations in microbiome analysis [35,36]. These pipelines represent fundamentally different analytical approaches [35,36]. QIIME2-based amplicon profiling relies on short-read denoising (DADA2) and naive Bayes classification of the 16S rRNA gene [37]. In contrast, Kraken2 performs k-mer-based classification of whole metagenome shotgun (WGS) reads against a reference database [38,39], while mOTUs uses marker genes extracted from reference genomes and metagenomic bins to quantify taxonomic abundance [39]. Applying Kraken2 and mOTUs to the same 19 WGS BioProjects enabled direct cross-pipeline comparison, whereas QIIME2 provided an independent view of aggregate genus-level patterns in the amplicon collection.

Despite the methodological differences inherent to taxonomic profiling [5,35,36,40], the analyses repeatedly recovered a common set of dominant cheese-associated bacterial genera. Quantitative concordance was strongest in the paired WGS comparison, with correlations of ρ = 0.513 for prevalence and ρ = 0.654 for mean relative abundance. Corresponding correlations were ρ = 0.361 and 0.482 for QIIME2–Kraken2 and ρ = 0.258 and 0.417 for QIIME2–mOTUs (Figure 5A; Supplementary Table S3). These results show both the reproducibility of dominant taxonomic patterns and the additional variation introduced when comparing independently assembled study collections. They support the use of public data for comparative analysis while demonstrating why quantitative estimates should remain linked to the profiling approach and analytical denominator used [40].

The species-level analysis extends comparative work on cheese microbiomes [9,19] by identifying taxa that satisfy the same widespread-occurrence criteria in two distinct WGS pipelines. Across 103 shared species retained after filtering, mean relative abundances were moderately correlated (ρ = 0.534, *p* = 6.36 × 10^−9^; Figure 5D). Agreement in the identification of widespread species, together with their leave-one-project-out stability, strengthens the evidence for their recurrence within the collection. At the same time, incomplete quantitative concordance is consistent with the influence of reference coverage and profiling procedures on abundance estimates [5,35,36]. This distinction is particularly important because strain-level and within-species diversity can remain substantial even when higher taxonomic levels appear highly concordant [13,40,41].

The present study complements, but differs in scope from, previous cheese microbiome investigations. Walsh et al. [19] established the value of meta-analysing cheese microbiomes for understanding quality-associated microbial patterns, whereas Liu et al. [9] developed a deeply resolved microbiome catalogue focused on Chinese traditional artisanal cheeses. Tsouggou et al. [36] synthesised recent methodological advances in shotgun metagenomics, while Sabater et al. [42] demonstrated the potential of metagenomics and multi-omics for authentication and traceability within a specific PDO cheese system. Our contribution is to integrate recurrent taxonomic patterns, country-associated variation, cross-pipeline comparisons, and metadata assessment within a standardized reanalysis of geographically distributed public datasets. This broader comparative perspective connects findings from individual cheese systems and identifies patterns that persist across heterogeneous studies. It also establishes explicit analytical definitions and sensitivity tests through which future datasets can be compared with the present collection.

The recurrent taxa and composition profiles identified here provide a basis for targeted investigations of fermentation performance, cheese quality, and microbial interactions. Integrative and functional approaches offer routes for connecting these taxonomic patterns to microbial activity and product characteristics [12,43,44,45,46,47]. In particular, the widespread species set provides candidates for comparative strain-level and functional studies, while the descriptive composition types can guide comparisons between communities with contrasting dominant bacteria [2,4,32,33,34]. The persistence of country-associated variation also motivates further investigation of provenance and traceability. Developing such applications will require testing predictive performance in independent samples within defined cheese types and production systems. More broadly, harmonized public datasets provide a foundation for integrating taxonomic profiles with functional metagenomics, metabolomics and other omics approaches, enabling future studies to connect recurrent community patterns with fermentation performance, sensory properties and microbial safety [12,14,44].

The scope of these findings is bounded by the sampling and metadata structure of public repositories, a central consideration in retrospective microbiome analysis [16]. European studies predominated in the geographically resolved collection of 89 BioProjects from 21 countries, and balanced subsampling cannot compensate for regions or cheese systems absent from the data. This matters given the diversity documented in broader food surveys and geographically focused cheese studies [9,16,27,28,29,48,49]. The collection also includes cheese-associated production stages rather than exclusively finished cheeses. Incomplete metadata prevented consistent adjustment for cheese type, milk treatment, starter use, ripening, and storage—variables relevant to interpreting variation across dairy studies [13,19,50,51]. Accordingly, the geographic association may include correlated production and study-level effects. Significant dispersion in the primary geographic analysis and in the composition-type subset further requires joint interpretation of PERMANOVA and PERMDISP. The sequential variance fractions are descriptive, and differences in the number of factor levels preclude treating their unadjusted R^2^ values as a direct ranking of biological importance [36,52,53].

A second limitation concerns analytical scope and technical heterogeneity. Standardized reprocessing cannot remove differences in DNA extraction, sequencing platforms, or amplicon regions [36]. Inconsistent primer and variable-region metadata prevented region-specific processing, and the workflow did not include a separate primer-removal step. The bacterial focus also leaves fungal components of cheese communities outside the principal analysis [52,53]. Widespread-species membership depends on detection and prevalence thresholds, while correlations across mean taxon abundances do not measure sample-level classification accuracy. These qualifications define how the reported patterns should be used. Likewise, the 49.9% residual fraction in the descriptive country–composition partition does not identify its causes; ecological variation, unmeasured production conditions, and technical differences are possible contributors rather than explanations established by the model [28,36].

The next step is to connect this comparative baseline with more complete production metadata and functional measurements. Consistent reporting of sample material and origin, cheese type, starter use, ripening and storage conditions, and laboratory protocols would allow future analyses to distinguish the components currently grouped within country and study effects [5,36]. Expanding geographic coverage while preserving transparent curation and reusable analytical workflows would further increase the value of public repositories as cumulative resources for food microbiome research [16,48].

Overall, this study identifies a stable set of widespread bacterial species and shows that country-associated community variation persists beyond differences in five dominant fermentation-associated genera. The coexistence of recurrent taxa and differentiated community profiles provides a coherent picture of cheese-associated microbial diversity across the public studies examined. By recovering these patterns through standardized reanalysis and testing their sensitivity to analytical choices and study representation, the work establishes a comparative foundation for investigating how shared fermentation ecology interacts with the diversity of cheese production systems.

## 4. Methods

### 4.1. Dataset Retrieval and BioProject Curation

Public cheese-associated microbiome datasets were identified in the NCBI Sequence Read Archive (SRA) using Entrez Direct (v26.0) [54] on 7 February 2026. The archived search query required the terms “cheese” and “metagenome” and excluded records associated with clearly unrelated host-associated, environmental, wastewater, urban and engineered microbial systems, including human, skin, oral, gut, wastewater, sewage, soil, marine, hospital and bioreactor-associated datasets. The exact archived search query, including all exclusion terms, is provided in the public reproducibility repository accompanying this study. The initial search returned 14,203 SRA records from 233 BioProjects. SRA RunInfo metadata were retrieved for all candidate records and integrated into a unified metadata table.

Run-level filtering was subsequently applied to retain records assigned to food- or dairy-associated metagenomic categories, including food fermentation metagenome, food metagenome, dairy metagenome, milk metagenome, cheese metagenome, food production metagenome and fermentation metagenome. These broad NCBI ScientificName categories were used as metadata filters and were not interpreted as direct descriptions of the physical sample material. To reduce inclusion of unrelated datasets, available SRA metadata fields describing the sample, study, design, BioProject and BioSample were screened using a case-insensitive blacklist of terms associated with host, environmental, wastewater, urban and engineered microbiomes. This metadata-based filtering retained 10,853 SRA records from 163 BioProjects for subsequent BioProject-level curation.

Only records with LibrarySource = METAGENOMIC and LibraryStrategy = AMPLICON or WGS were retained for BioProject-level curation. BioProjects containing at least five eligible runs and with ≥80% of runs matching the predefined food- or dairy-associated categories were automatically retained, whereas BioProjects with ≤20% matching runs were excluded. BioProjects containing fewer than five eligible runs or intermediate proportions were classified as ambiguous and manually reviewed using the available BioProject and sample metadata. Twenty-nine BioProjects required manual review, of which 20 were retained and nine were excluded. The final curated dataset, comprising automatically retained and manually accepted BioProjects, contained 8910 SRA records from 112 unique BioProjects (8100 amplicon records from 92 BioProjects and 810 WGS records from 21 BioProjects); one BioProject contained both sequencing strategies. Run-level inclusion and processing status are provided in Supplementary Table S11.

The study was designed to represent public cheese-associated microbiome datasets, rather than exclusively samples collected from the final cheese matrix. This distinction reflects the organization of public SRA data, in which cheese-related studies may contain samples representing cheese, cheese surfaces, curd, rind or other components and stages of cheese production, while individual records may be deposited under broad categories such as food production metagenome, dairy metagenome or milk metagenome. Importantly, these broad categories alone were not sufficient for dataset discovery: the initial SRA search explicitly required an association with “cheese”.

Sequencing data were retrieved as FASTQ files, primarily through synchronized records available from the European Nucleotide Archive (ENA) via HTTPS. The SRA Toolkit (prefetch and fasterq-dump) provided an alternative retrieval route, with fasterq-dump configured to separate paired reads and omit technical reads. FASTQ files were validated after retrieval and sequencing layout was identified as paired-end or single-end. No additional global read trimming, primer removal or host-read depletion was performed before the pipeline-specific taxonomic processing described below. Geographic metadata were assigned independently of sequencing-center location. Sample-origin country was extracted from geographic attributes reported in the corresponding NCBI BioSample records. BioProjects were classified as single-country only when the available BioSample metadata consistently indicated one sample-origin country; BioProjects containing samples from multiple countries were retained as multi-country projects and were not assigned to a single country. BioProjects lacking sufficient BioSample geographic information were classified as unresolved. Sequencing-center location was not used as a proxy for sample origin. For the geographically resolved dataset used in Figure 1, sample-origin information was available for 7592 retained SRA records belonging to 89 unique successfully profiled BioProjects across 21 countries. Two WGS BioProjects contained samples from multiple countries and were therefore represented in each corresponding geographic group but excluded from single-country community analyses. The resulting geographic assignments and their metadata status are provided in Supplementary Table S4.

### 4.2. Taxonomic Profiling of Amplicon Datasets

Amplicon datasets were processed using a standardized QIIME2 2026.1-based workflow [55]. Only runs classified as amplicon datasets in the curated metadata table and successfully retrieved in FASTQ format were included. Sequencing layout was automatically identified as paired-end or single-end prior to import into QIIME2 using Phred33 quality encoding.

Sequences were denoised using DADA2 (v1.30.0) [37] within QIIME2, generating representative sequences and feature tables for each sequencing run. Paired-end datasets were processed using qiime dada2 denoise-paired, whereas single-end datasets were processed using qiime dada2 denoise-single. Forward and reverse truncation lengths were set to 0, with the remaining DADA2 parameters left at the QIIME2 2026.1 defaults. No separate primer-removal step or sequencing-region-specific processing was applied. Primer sequences and targeted 16S rRNA variable regions were not consistently available in the harmonized SRA metadata across the public datasets and were therefore not used to stratify processing. Consequently, amplicon datasets targeting different 16S rRNA regions were processed using the same standardized workflow rather than region-specific parameter sets.

Taxonomic classification was performed using the SILVA 138 [56] 99% Naive Bayes classifier, and taxonomic assignments were collapsed to the genus level. Per-run genus-level abundance tables were exported and merged into a unified amplicon taxonomic count matrix with taxa as rows and SRA runs as columns. Total retained reads and unassigned reads were recorded and used for downstream quality control, prevalence, abundance and community-composition analyses. Of the 8100 amplicon SRA records in the curated dataset, taxonomic profiles were successfully generated for 7649 runs representing 88 BioProjects. After harmonization, removal of non-informative taxonomic entries and exclusion of samples with zero retained taxonomic abundance, the final amplicon analytical matrix comprised 7530 SRA runs from 85 unique BioProjects. Run-level processing status is provided in Supplementary Table S11.

### 4.3. Taxonomic Profiling of Whole-Genome Shotgun Datasets

Whole-genome shotgun datasets were analyzed using two independent taxonomic profiling approaches, Kraken2 (v 2.1.2) [38] and mOTUs (v4.0.4) [39]. Only runs classified as WGS datasets in the curated metadata table and available in FASTQ format were included. Sequencing layout was automatically identified as paired-end or single-end for each run.

Kraken2 profiling was performed using a locally installed nucleotide reference database built in September 2024 and comprising bacterial, archaeal, viral, plasmid, human, and UniVec_Core sequence libraries. Database inspection indicated 50,084 taxonomic nodes, with k = 35 and l = 31. Paired-end reads were analyzed using the paired option, whereas single-end reads were analyzed directly; 28 processing threads were used, and the remaining classification parameters were left at their default settings. Kraken2 reports were retained with taxonomic names, ranks, NCBI taxon identifiers, and abundance information. For the final harmonized matrices, entries assigned specifically to genus (G) or species (S) rank were extracted, and the percentage of reads assigned to each taxon and its descendants, as reported in the standard Kraken2 report, was converted to proportional abundance. Genus- and species-level matrices were constructed independently using entries at the corresponding taxonomic rank.

The same WGS datasets were independently profiled using mOTUs v4. Paired-end datasets were supplied using the forward and reverse read options and single-end datasets using the single-read option, with 28 processing threads and the remaining profiling parameters left at their defaults. The run-specific taxon count field reported by mOTUs was extracted to construct abundance matrices and was subsequently normalized within each run to relative abundance during downstream harmonization. Genus- and species-level profiles were retained separately for the corresponding downstream analyses.

Kraken2 and mOTUs outputs were retained separately throughout the analysis. Of the 810 WGS SRA runs in the final curated metadata dataset, Kraken2 profiles were available for 634 runs representing 20 BioProjects, whereas mOTUs profiles were available for 618 runs representing 19 BioProjects. A total of 618 runs from 19 BioProjects had profiles from both pipelines and constituted the directly paired Kraken2–mOTUs comparison set. All 618 runs successfully profiled by mOTUs also had corresponding Kraken2 profiles; therefore, these 618 runs from 19 BioProjects constituted the directly paired Kraken2–mOTUs comparison set. Run-level processing status and pipeline-specific profile availability are provided in Supplementary Table S11.

### 4.4. Bioinformatic and Statistical Analysis

All downstream analyses were performed using custom scripts in R (v. 4.5.2) and Python (v 3.14). The workflow was designed to reprocess public cheese microbiome datasets in a standardized manner independently of the original study, sequencing project or taxonomic profiling method. To reduce pseudoreplication caused by multiple sequencing runs from the same study, BioProject was treated as the independent analytical unit throughout the comparative analyses. Unless otherwise stated, prevalence estimates, abundance summaries, community-level statistics, concordance analyses and visualizations were generated from BioProject-level abundance matrices.

Taxonomic count matrices generated by QIIME2, Kraken2 and mOTUs were imported into R, standardized and filtered prior to analysis. Duplicated SRA accession columns were collapsed, and non-informative entries, including unassigned taxa, unclassified records, root-level classifications, empty taxonomic strings and technical placeholders, were removed. Host-associated taxa were excluded at the collapsed taxonomic level. Count matrices were converted to relative abundance matrices by normalizing each sequencing run to its total retained taxonomic count. Samples with zero total abundance after filtering were excluded. After these harmonization and filtering steps, the genus-level analytical matrices contained 85 unique BioProjects for QIIME2, 20 for Kraken2 and 19 for mOTUs.

For each BioProject, relative abundances of individual taxa were averaged across associated SRA runs to generate a representative BioProject-level profile for each analytical pipeline. Amplicon data were analyzed at the genus level, whereas WGS data were analyzed at both genus and species levels. Taxon prevalence was calculated as the proportion of BioProjects in which a given taxon reached a relative abundance of at least 0.01%. Mean and median BioProject-level relative abundances were calculated independently for each pipeline.

For genus-level visualization, genera were ranked according to their prevalence and mean relative abundance across the three analytical pipelines. The genera displayed in Figure 3 represent prevalent cheese-associated, surface-ripening-associated and environmental bacterial genera selected for comparative visualization and should not be interpreted as a formally defined universal core microbiome.

Cross-pipeline genus-level concordance was evaluated separately for each pair of analytical pipelines. Kraken2 and mOTUs were applied to the same WGS collection and therefore provided a direct comparison of taxonomic profiling methods. The paired Kraken2–mOTUs comparison was restricted to the 19 BioProjects successfully profiled by both pipelines. In contrast, QIIME2 was applied to the independently assembled amplicon collection; consequently, QIIME2–Kraken2 and QIIME2–mOTUs comparisons were interpreted as concordance of aggregate taxonomic patterns across different public study collections rather than direct method-to-method agreement. Pairwise Spearman rank correlations were calculated for BioProject prevalence and mean relative abundance. Similarity in detected genus sets was additionally quantified using the Jaccard index. To reduce the influence of rare taxa, filtered concordance analyses retained genera that met both of the following criteria: prevalence ≥10% in at least one of the two compared pipelines and mean BioProject-level relative abundance ≥0.01% in at least one of the two compared pipelines.

For WGS species-level analyses, Kraken2 and mOTUs profiles were evaluated independently before identifying species satisfying the criteria in both pipelines. After collapsing duplicated BioProject entries, the analytical denominators were 20 unique BioProjects for Kraken2 and 19 unique BioProjects for mOTUs. Species were considered detected within a BioProject at a relative abundance of at least 0.01%. A species was classified as widespread when it was detected in at least 50% of BioProjects independently in both Kraken2 and mOTUs and had a mean BioProject-level relative abundance of at least 0.01% in both pipelines. Species detected in at least 75% of BioProjects in both pipelines were additionally classified as highly widespread. Species occurring in at least 10% but less than 50% of BioProjects in at least one pipeline were treated as recurrent taxa rather than members of the widespread species set.

Robustness of the WGS species-level definition was evaluated by varying both the BioProject-level detection threshold and the required prevalence threshold. Detection thresholds of 0, 0.001%, 0.01% and 0.1% relative abundance were evaluated together with prevalence thresholds of 10%, 25%, 50%, 75% and 90%, while retaining a mean abundance criterion of 0.01%. Stability of the primary widespread species set was further assessed using leave-one-BioProject-out analysis, in which individual WGS BioProjects were sequentially excluded and the prevalence criteria recalculated. Species-level classifications, threshold-sensitivity results and leave-one-BioProject-out stability are reported in Supplementary Tables S8–S10.

Species richness within dominant genera was calculated separately for Kraken2 and mOTUs. Quantitative abundance concordance between the two WGS pipelines was evaluated across shared species retained after prevalence and abundance filtering using the same prevalence-and-abundance filtering principle applied to the concordance analyses: species were retained when prevalence was ≥10% in at least one WGS pipeline and mean BioProject-level relative abundance was ≥0.01% in at least one WGS pipeline. This filtering retained 103 of the 448 species shared between Kraken2 and mOTUs. Spearman rank correlation was then calculated between the across-BioProject mean relative abundances estimated independently by the two pipelines; this analysis therefore assessed concordance across species rather than sample-level classification accuracy.

Composition-associated community structure was evaluated using the relative abundances of *Lactococcus*, *Streptococcus*, *Lacticaseibacillus*, *Lactobacillus* and *Leuconostoc.* BioProjects with a non-zero cumulative abundance of these genera were transformed to within-BioProject five-genus composition profiles. This resulted in 68 BioProjects for composition-associated analyses. K-means clustering was evaluated across k = 2–10, with repeated random initializations used to assess the dependence of the partition on the number of clusters and initialization. A five-group solution was retained as a descriptive representation of contrasting community compositions and was not selected as a uniquely optimal clustering solution. It was not interpreted as evidence of five discrete starter-culture or production-technology categories. These groups are therefore referred to as descriptive composition types.

Microbial community structure was evaluated using BioProject-level relative abundance matrices. Hellinger transformation was applied before calculation of Bray–Curtis dissimilarities. PERMANOVA was performed using vegan::adonis2 with 999 permutations. Geographic analyses were restricted to BioProjects assigned to a single BioSample-reported sample-origin country. The primary geographic analysis included 73 amplicon BioProjects representing 20 countries. Homogeneity of multivariate dispersion was assessed using vegan::betadisper followed by permutation testing. Because significant PERMDISP indicates heterogeneity in within-group dispersion, PERMANOVA results were interpreted together with the corresponding dispersion tests rather than as evidence of differences in group centroids alone.

Associations of country and descriptive composition type with community structure were evaluated using single-factor and sequential PERMANOVA models. For multi-term models, terms were evaluated sequentially (by = “terms”), and both variable orders were examined to quantify the additional variation associated with each variable after the other had been entered into the model. A descriptive partition of variation into country-associated, composition-type-associated, shared and residual components was derived from these sequential models; these components were interpreted as statistical associations rather than independent causal effects. Because sequential sums of squares depend on term order, the resulting fractions were used descriptively and were not interpreted as uniquely identifiable causal components of community variation.

Robustness of the geographic association was evaluated using four complementary sensitivity analyses. First, PERMANOVA and PERMDISP were repeated after restricting the dataset to countries represented by at least two BioProjects (63 BioProjects from 10 countries). Second, balanced subsampling was performed over 1000 iterations by randomly selecting three BioProjects from each of eight countries represented by at least three BioProjects, yielding 24 BioProjects per iteration. PERMANOVA and PERMDISP were recalculated independently for each iteration, and the distribution of country-associated R^2^ values and permutation-test results was summarized across iterations. Third, the geographic analysis was repeated after removing *Lactococcus*, *Streptococcus*, *Lacticaseibacillus*, *Lactobacillus* and *Leuconostoc* from the community matrix before transformation and dissimilarity calculation. Fourth, an additional sequential PERMANOVA model treated the relative contributions of these genera as continuous composition covariates rather than discrete composition types. The latter analysis was restricted to the 68 BioProjects with non-zero cumulative abundance of the five genera. Full geographic assignments and statistical outputs are provided in Supplementary Tables S4–S7.

Additional descriptive analyses included BioProject size distributions, temporal deposition trends, sequencing platform summaries, library strategy summaries, geographic summaries and matrix-versus-metadata consistency checks. Geographic summaries were based on sample-origin information reported in NCBI BioSample metadata rather than sequencing-center location. BioProjects with samples assigned to multiple countries or without sufficient sample-origin information were not assigned to a single country for country-level community analyses. Unless otherwise stated, statistical tests were two-sided, and *p* < 0.05 was considered statistically significant.

All scripts used for dataset curation, taxonomic harmonization, statistical analyses, sensitivity analyses and figure generation, together with processed BioProject-level matrices and analysis settings, are available in the public GitHub repository: https://github.com/Djohn20s/cheese-microbiome-global-reanalysis (accessed on 29 October 2026).

## Figures and Tables

**Table 1:** Filtering workflow summary.

Metric	SRA	Bioprojects
Initial NCBI query	14,203	233
Metadata-based filtering	10,853	163
Automatic and manual BioProject curation	8910	112
WGS subset	810	21 *
Amplicon subset	8100	92 *

* One BioProject contained both amplicon and WGS datasets and was therefore included in both sequencing subsets.

## Data Availability

The sequencing datasets analyzed in this study are publicly available from the NCBI Sequence Read Archive (SRA) under the BioProject accessions listed in Supplementary Table S1. All analyses were performed using publicly available sequencing data. The curated metadata tables, processed abundance matrices, source data underlying the figures, analysis settings, and scripts used for dataset curation, taxonomic harmonization, statistical analyses, sensitivity analyses, and figure generation are publicly available in the GitHub repository: https://github.com/Djohn20s/cheese-microbiome-global-reanalysis (accessed on 29 October 2026). Run-level inclusion and processing status are provided in Supplementary Table S11. The complete list of BioProjects included in the final curated dataset is provided in Supplementary Table S1.

## Supplementary Materials

The following supporting information can be downloaded at: https://www.mdpi.com/article/10.3390/ijms27219798/s1.
